# Development of an interprofessional task-based learning program in the field of occupational health: a content validity study

**DOI:** 10.1186/s12909-022-03997-1

**Published:** 2023-01-07

**Authors:** Fatemeh Keshmiri, Amir Houshang Mehrparvar

**Affiliations:** 1 Education Development Center, Shahid Sadoughi University of Medical Sciences, Yazd, Iran; 2 Industrial Diseases Research Center, Shahid Sadoughi University of Medical Sciences, Yazd, Iran

**Keywords:** Task-based learning, Interprofessional learning, Occupational health services, Occupational medicine, Industrial hygiene

## Abstract

**Objective:**

One of the duties of the educational system is to provide situations in which students learn the tasks corresponding to their future careers in an interprofessional team. This study was designed to develop an interprofessional task-based training program.

**Methods:**

This was a curriculum development study conducted by content validity methodology in two stages: 1) ‘framework development’ which resulted in the creation of the framework items; and 2) ‘evaluation of the framework’ (judgment and quantification). The first stage consisted of task identification, generation of sub-tasks, and assimilation of items into a usable format. The second stage consisted of the judgment –quantification of the content validity of items and the framework. After that, the framework of the tasks of the occupational health team was finalized in the expert panel. After explaining the tasks, a matrix for task-expected roles in the occupational health team and a matrix for task-required skills to perform each task were developed. The next step determined the appropriate teaching and assessment methods for each task. Finally, an expert panel reviewed and approved the components of the interprofessional task-based training program.

**Results:**

Integrating the interprofessional education strategy with task-based learning was considered innovative in occupational health team training. In the development stage, 48 items were extracted, and then 35 tasks were generated in the step of identification of tasks. In the second step, 174 sub-tasks were developed. The tasks and sub-tasks were categorized into seven areas. After the stage of evaluation of the framework, 33 tasks were categorized into seven main areas, including "assessment and identification of workplace hazards" (*n* = 10), "control of occupational hazards" (*n* = 4), "determining the appropriate job position for each person" (*n* = 3), "occupational health examinations" (*n* = 6), "management of occupational/work-related diseases" (*n* = 5), "inter-organizational and inter-disciplinary relations, and legal judgment" (*n* = 3) and "education and scholarship in occupational health services" (*n* = 2).

**Conclusion:**

The results of the present study can be used in developing the use of the interprofessional strategy and task-based training as two appropriate strategies for the purposeful development of learners' abilities in the fields involved in providing occupational health services in their future careers.

## Introduction

Interprofessional collaboration has been introduced as a necessity in providing occupational health services [1]. Occupational health services, including workplace assessment, counseling, diagnosis and treatment of occupational diseases, and follow-up services for employees at the beginning of employment, during employment, and after the emergence of occupational diseases is provided by a team [2, 3]. Understanding the role of members of the occupational health team and establishing collaborative relationships between different disciplines is very important in providing high-quality and safe services [1]. In the occupational health team, individuals from different professions consisting of occupational medicine specialists, occupational hygienists, general practitioners, ergonomists, safety experts, and in some countries, occupational nurses are brought together, and it is necessary to use interprofessional collaboration to provide occupational health services. The occupational health team needs to work together to reduce workplace hazards, improve ergonomic problems, maintain workability, prevent occupational diseases, and improve the psychological well-being of employees in the workplace [1]. Occupational medicine physicians and occupational hygienists are considered the key members of the team and they complete each other’ roles in providing occupational health services with some overlapping tasks, so they need each other for providing efficient services which require interprofessional collaboration [4]. Occupational hygiene professionals play an essential role in gathering information for occupational medicine specialists by identifying, determining, and controlling occupational exposures to chemical, biological, ergonomic, psychological, and physical hazards in the workplace. The occupational medicine specialist uses occupational exposure assessments performed by occupational hygiene professionals to diagnose and treat workers appropriately in a participatory decision-making manner [4]. Interprofessional practice in occupational health occurs when practitioners from two or more professions work together with a common purpose, commitment, and mutual respect [5]. The use of interprofessional collaborative competencies including effective communication, collaboration, recognition of members’ roles and responsibilities and team functions, leadership, and conflict management [4, 6–10] are essential in providing occupational health services.

The critical tasks of the team members in providing occupational health services are defined as follows: 1) Leading, supporting, and providing professional and technical advice to workers and employers; 2) Development of standards, reporting systems, and policies required to promote occupational health; 3) Occupational injury assessment and management; 4) Identification of occupational hazards and prevention of occupational diseases; 5) Promoting the general health of employees [11]. Many of these tasks are not included in the curriculum of occupational health team members, especially occupational medicine specialists. An occupational physician must be able to work with specialists in other related fields (occupational hygiene, safety, and ergonomics) and has a proper relationship with workers, employers, companies, and institutions related to occupational health services, and other organizations such as the labor office, forensic authorities, and insurance organizations. On the other hand, tasks such as promoting public and environmental health are among the sub-tasks of these people [12–14].

Current training in various fields involved in occupational health services is provided in a uni-professional strategy. Many studies have shown insufficient efficiency of this training strategy, which can be due to the neglect of some competencies including soft skills such as communication, teamwork, interprofessional collaboration, and professionalism in formal and informal education of learners which may ultimately affect the occupational health care system [15].

A survey conducted in Italy on the characteristics of professional activities and performance of occupational physicians addressed working relationships with other related disciplines, workers, and employers. Many participants in this study believed that various changes should be made to the curriculum to meet the real needs of occupational medicine specialists in practical work. In this study, there was a significant difference between the capabilities of different people, which indicates a possible defect and difference in the education of individuals whose training is not focused on learning based on their tasks [16]. The problems of occupational health services in Iran have pointed out the shortcomings of educational curricula and the need for change [11].

Interprofessional education is an occasion that members or students of two or more professions learn about, with, and from each other, to improve collaboration, and the quality of care and services [9, 17, 18]. It involves teachers and learners from two or more related professions who provide a collaborative learning environment. An interprofessional team consists of people from various professions with specialized knowledge, skills, and abilities, but with a common goal [6]. In this method, all team members attach importance to joint decision-making [6]. Interaction and interprofessional practice are considered essential in developing collaboration in health promotion, prevention, treatment, and rehabilitation services [2]. This service provision strategy promotes the health of individuals and communities in various fields and was supported by World Health Organization [18].

In task-based learning (TBL), the focus is on learning a set of tasks performed by a healthcare provider in a real situation [19, 20]. In this method, learning takes place around performing tasks and achieving results so that learners try to understand the tasks themselves and the underlying concepts and mechanisms of each task [21]. TBL is a learning method for workplace-based training that provides a reasonable opportunity for problem-based learning and integrated multidisciplinary teaching [22].

Integrating strategies of interprofessional education and task-based learning in the educational program provides an excellent opportunity for the students to be familiar with the tasks and work issues similar to the real environment and learn how to manage them in an interprofessional team [23].

One of the methods to develop an educational program is the content validity methodology. This method can provide evidence about how relevant the elements of a framework are and to what degree they represent a purposeful structure for a specific goal [24, 25]. Face validity and logical validity are the two main components of content validity. The former indicates the validity of a measure “on its face.”, but the latter needs a process such as using an expert panel to evaluate the content validity of a measure [26]. Almanasreh et al. suggested a process for the content validity method consisting of three stages: development, judgment, quantifying and revising and reconstruction [24]. Clarity and representativeness of different items of a measure can be identified by a content validity study. Rubio et al. proposed the following stages for a content validity study: forming an expert panel and then evaluating the quantitative and qualitative indices of validity [26].

This study was designed to develop an interprofessional task-based training program by integrating the strategies of interprofessional education and task-based learning for learners in disciplines who provide occupational health services.

## Materials and methods

**Fig. 1:**
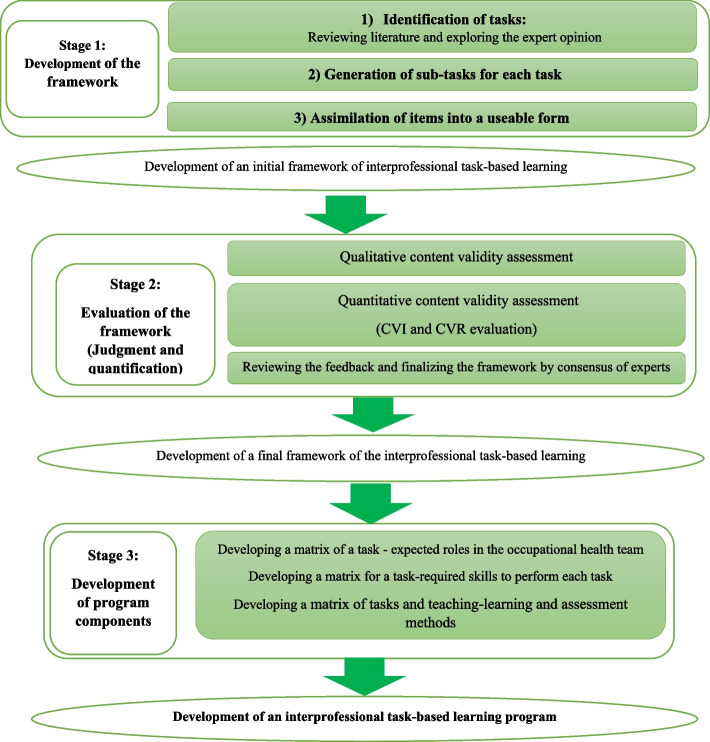
Flow chart of study steps

This was a curriculum development study conducted by content validity methodology. The content validity study was used based on the steps described by Lynn [27]. This study was performed in two stages, including 1) the development stage and 2) the evaluation stage (judgment and quantification of the framework and its items) (Fig. 1).

In the current study, to develop an interprofessional task-based program, some general principles were considered.

### General principles in the development of interprofessional task-based learning program

- **Task-based learning** (using the principles of task-based learning): In this strategy, the learners perform the defined task in the best way to prepare for their future careers [21]. The task-based learning provides a situation for the learners to face their professional problems/issues and learn about their tasks and the method of solving the issues in a real environment. The study focuses on the task that members of the occupational health team require to conduct through interprofessional collaboration.
- **Interprofessional education** (using interprofessional education as a main educational strategy in the program): Interprofessional education provides a situation in which learners in different disciplines learn how to work in an interprofessional team [6]. To achieve the interprofessional education strategy, the members of different disciplines involving in the provision of occupational health services should participate in all teaching and learning methods.
- **Interprofessional team**: “The team is composed of members/learners from different health professions who have specialized knowledge, skills, and abilities” [6]. In the present study, the knowledge, skills, and attitudes that may result in interprofessional behaviors and competence were developed.

### Stage one: development of a framework

The stage of development consisted of 1) identification of tasks, 2) generation of sub-tasks for all tasks and 3) assimilation of items into a usable form [27].

1) *Identification of tasks*: In this step review of the literature and exploring, expert opinions were done. At this stage, a review was conducted to identify the interprofessional task activities of the occupational health team. The literature on the tasks, competencies and expected outcomes of the members of an occupational health team in an interprofessional collaboration was reviewed. This step reviewed existing frameworks, curricula, and programs that addressed interprofessional collaboration in the occupational health field. The competency frameworks of interprofessional collaboration were reviewed as well [28]. In addition, the opinions of experts regarding the tasks of the occupational health team were explored.

To do this, the participants were selected by purposeful sampling. Participants were graduates and faculty members who had worked in an occupational health team for at least three years at five universities in Iran. After identifying participants (*n* = 47), a Delphi technique was implemented in electronic form. First, a cover letter was sent to the participants to clarify the aims of the research, the expectations, the timetable, and the relationship channels (e-mail, synchronized virtual meetings, and social networks). The participants were asked to list the expected tasks of occupational team members in different disciplines (occupational medicine, occupational hygiene, and ergonomics). The opinions of participants were collected and categorized in four Delphi rounds. After the fourth round, no new task emerged.

After that, the results of the literature review and the opinion of experts were appraised in expert panel meetings with team members from different fields of occupational health and an expert in medical education (*n* = 5). The items were converted to ‘tasks' according to the criteria of the task-based learning strategy [19, 20].

2) *Generation of sub-tasks for each task*: Based on the task-based learning strategy, sub-tasks were formulated by considering the skills, knowledge, and attitudes required to perform each task. The sub-tasks were generated in the meeting of expert panels where the experts in different disciplines participated.

3) *Assimilation of tasks into a usable form*: in this stage, the tasks and sub-tasks with similar themes were classified into seven areas.

### Stage Two: Evaluation of the framework (judgment and quantification).

The evaluation of the framework included the judgment quantification of the content validity of items and the framework [27]. In this stage, the qualitative and quantitative content validity of the formulated tasks was assessed from the viewpoints of various stakeholders, including faculty members and graduates of the disciplines providing occupational health services (*n* = 16).

### 1) Qualitative content validity assessment

IN this step, a qualitative content validity assessment form was sent to the experts (*n* = 16). The experts in the session differed from the participants who attended the development stage. After two weeks, the comments, and submitted feedback regarding the tasks, subtasks, and framework were collected. After that, the experts (*n* = 5) reviewed the submitted feedback from the participants. The items and framework were finalized in the step by the consensus among experts.

### 2) Quantitative content validity assessment

The quantitative indicators of content validity were examined. Two content validity indicators, "Content Validity Ratio" (CVR) and "Content Validity Index" (CVI), were used [24, 26, 29, 30].

For CVR measurement, a group of experts is employed to assess the appropriateness of framework items that reflect the domain construct on a three-point scale: [1] essential, [2] useful but not essential, and [3] not essential. The minimum value of the content validity ratio was determined based on Lawshe’s table [31].

CVI assesses the content relevancy of each item with the content of the framework [32]. For CVI, each competency criterion of "relevance" was assessed using a four-point Likert scale [33]. In this study, the item-level content validity index (I-CVI) was examined for each task, and the scale-level content validity index (S-CVI / Ave) which is an average of item-level content validity indices for the framework (all items) was calculated [33]. When there are five or fewer experts, I-CVI is recommended to be 1.00, while a minimum I-CVI of 0.78 is recommended when there are more than five experts [30]. In the study, the minimum acceptable CVR according to Lawshe’s table was determined to be 0.49 [34]. The results were reviewed in the expert panel, and proposed amendments and quantitative validity indicators of each task were discussed. At this stage, the tasks and the framework were finalized by consensus among experts.

### Development of program components

After explaining the tasks, according to the task-based learning strategy [19, 20], three matrices were developed, including 1) a matrix of task-expected roles in the occupational health team; 2) a matrix for task-required skills to perform each task; 3) a matrix of the teaching and appropriate assessment methods of the tasks. Finally, the experts reviewed and approved the components of the interprofessional task-based training program.

In total, 25 expert panel sessions were held over 14 months to develop the task-based interprofessional training program for occupational health services.

## Results

A total of 68 faculty members and graduates of different disciplines involved in providing occupational health services participated in the study.

- Five experts participated in the expert panel meetings including an expert in medical education and two experts in occupational medicine and an expert in occupational hygiene and an expert in ergonomics. Their mean age was 40 ± 13 years.
- Forty-seven faculty members and graduates in occupational medicine (25 people, 53.19%) and occupational hygiene (16 people, 34.04%), and ergonomics (6 people, 12.76%) participated in Delphi rounds in the development stage. Their mean age was 46 ± 9.23 years.
- In the evaluation of the framework stage, 16 specialists and faculty members participated. Of these, nine were males (56.25%), and seven were females (43.75%). Their mean age was 47.66 ± 8.73 years.

In the development stage, 48 items were extracted and 35 tasks were generated in the step of identification of tasks. In the second step (*generation of sub-tasks for each task*), 174 sub-tasks were developed. The tasks and sub-tasks were categorized into seven areas.

In the evaluation of the framework stage, three tasks were merged according to the submitted feedback in the qualitative content validity step. Moreover, 12 sub-tasks were suggested to be removed and/or merged. Finally, the content validity of 33 tasks was assessed qualitatively.

The results showed that the CVR value for all items was higher than the minimum acceptable value, so all items were kept in the framework. According to the I-CVI index, the CVI values of all competencies were above 0.79 and were maintained in the framework. Quantitative content validity results are reported in Table 1. Quantitative validity test results for the framework were confirmed by S-CVI / Ave = 0.78 (scale-level content validity index).

**Table 1:** Results of quantitative content validity assessment

	Content Validity Ratio	Content Validity Index
**Axis: Identifying and assessing the risk of workplace hazards**		
1.To carry out an initial assessment of the work environment in the occupational health team (walkthrough survey)	0.83	1
2.To design a plan for environmental quantitative exposure measurements based on the results of the initial assessment	0.83	1
3.To design a plan for personal quantitative exposure measurements based on the results of the initial assessment	0.83	1
4.To measure the psychological hazards of the workplace	0.83	1
5.To measure the ergonomic hazards of the workplace	0.83	1
6.To develop an occupational exposure matrix of employees in the occupational health team based on the results of the measurements	0.66	1
7.To prepare a report of assessments and measurements of workplace exposures in cooperation with the members of the occupational health team	0.83	1
8.To interpret the results of the measurements and present them to different stakeholders	0.83	1
9.To identify occupations with risk level 1 (carcinogens and incurable diseases) and provide recommendations based on improving occupational health indicators in these occupations	0.83	0.91
10.To identify the hazards of high-standard jobs	1	0.91
**Axis: Controlling occupational hazards**		
11.To plan for controlling occupational hazards based on prioritization	0.83	0.91
12.To manage occupational biologic hazards by the occupational health team	0.66	0.91
13.To manage occupational psychologic hazards by the occupational health team	0.66	0.91
14.To manage occupational ergonomic hazards by the occupational health team	1	0.91
**Axis: determining the appropriate job position for each person**		
15.To determine the appropriate job position for employees based on demographic characteristics, individual capabilities, occupational hazards, and underlying diseases	0.83	1
16.To estimate the physical workload according to the reports of the occupational health team and find the appropriate person for this job	0.56	0.91
17.To estimate the mental workload according to the reports of the occupational health team and find the appropriate person for this job	0.56	0.91
**Axis: Occupational health examinations**		
18.To design occupational health examinations based on the reports of the occupational health team	0.56	1
19.To conduct occupational health examinations based on the designed examinations	0.56	1
20.To make appropriate decisions about the employment of the individuals based on the conducted evaluations	0.66	1
21.To apply all relevant national and international standards and requirements in the provision of occupational health services	1	1
22.To report the results of occupational health assessments to the relevant authorities	1	0.91
23.To provide non-occupational disease prevention services to employees	0.83	1
**Axis: Managing work-related/occupational diseases**		
24.To use appropriate methods to diagnose occupational diseases	1	0.91
25.To use appropriate methods to treat occupational diseases	0.66	1
26.To use appropriate methods for rehabilitation and early return to work of employees	0.66	1
27.To determine the relationship between workplace exposures and occupational diseases	0.83	0.83
28.To register and report occupational diseases according to national and international guidelines	1	1
**Axis: Inter-organizational and inter-disciplinary relations and legal judgment**		
29.To cooperate with external organizations (forensic medicine, insurance organizations, medical council, the judiciary, and labor office	0.66	0.91
30.To seek advice from specialists in other fields for proper diagnosis and treatment of occupational and non-occupational diseases only when necessary	0.66	1
31.To collaborate in monitoring the provision of occupational health services	0.83	1
**Axis: Education and scholarship in providing occupational health services**		
32.To collaborate in educating different stakeholders	0.83	0.91
33.To apply the principles of informed decision-making in scholarly and professional activities	0.83	0.91

Finally, 33 tasks were categorized into seven main areas, including "assessment and identification of workplace hazards" (*n* = 10), "control of occupational hazards" (*n* = 4), "determining the appropriate job position for each person" (*n* = 3), "occupational health examinations" (*n* = 6), "management of occupational/work-related diseases" (*n* = 5), "inter-organizational, inter-disciplinary relations and legal judgment" (*n* = 3) and "education and scholarship in occupational health services" (*n* = 2).

The tasks and sub-tasks of the occupational health team are reported in Table 2.

**Table 2:** Task framework of the occupational health team in task-based interprofessional training

Axis: Identifying and assessing the risk of workplace hazards (exposure risk assessment)
**To carry out an initial assessment of the work environment in the form of an occupational health team (walkthrough survey)** 1.To perform an observational assessment of the entire job process 2.To determine different workstations 3.To identify occupational exposures of each workstation by type of exposure 4.To identify chemical exposures in the workplace 5.To identify physical exposures in the workplace 6.To identify ergonomic exposures in the workplace 7.To identify biological exposures in the workplace 8.To identify the causes of occupational infections in the workplace 9.To identify psychological exposures in the workplace 10.10.To identify the causes of job stress in the workplace 11.To determine the high-risk points of the work environment in terms of occupational exposures 12.To develop a job ID for each job title 13.To develop a workshop ID for each workshop
**To design a plan for quantitative environmental exposure measurements based on the initial assessment results** 14.To design an environmental measurement plan for the exposures based on the initial assessment results 15.To measure the exposure to chemical agents in the workplace with standard methods 16.To measure the exposure to noise by standard methods using a sound level meter 17.To measure exposure to ionizing radiation by standard methods 18.To measure exposure to non-ionizing radiation by standard methods 19.To measure exposure to vibration by standard methods 20.To measure exposure to a high temperature by standard methods 21.To measure exposure to low temperature by standard methods
**To design a plan for personal quantitative exposure measurements based on the initial assessment results** 22.To design a personal measurement plan for the exposures based on the initial assessment results 23.To measure the individual exposure to chemical agents in the workplace with standard methods 24.To measure personal exposure to noise by standard methods using a personal dosimeter 25.To measure personal exposure to ionizing radiation by standard methods
**To measure the psychological hazards of the workplace** 26.To determine the mental workload of the workplace based on primary assessments 27.To determine factors causing job stress based on primary assessments
**To measure ergonomic hazards of the workplace** 28.To measure identified physical ergonomic hazards of the workplace using standard methods 29.To perform posture analysis of the workers during work 30.To determine the risk of occupational musculoskeletal disorders based on the results of posture analysis 31.To determine the priorities that need correction in the workplace based on the results of the posture analysis 32.To estimate the physical workload of different jobs based on the evaluations performed 33.To categorize jobs based on the required energy to very light, light, medium, heavy, and very heavy classes 34.To measure the identified cognitive ergonomic factors in the workplace
**To compile an occupational exposure matrix in the occupational health team based on the measurements** 35.To prioritize occupational exposures based on severity, importance, and risk 36.To draw job exposure matrix using standard methods
**To prepare a report of assessments and measurements of workplace exposures in cooperation with the members of the occupational health team** .37.To compile the initial evaluation report and measurements separately for different workstations
**To interpret the results of the measurements and present them to different stakeholders** 38.To present the results in an understandable language to the employer, insurance companies, health centers, and occupational examination centers
**To identify occupations with risk level 1** 39.To identify occupational carcinogens in different workstations based on the report of workplace assessments 40.To identify the causes of incurable diseases in different workstations based on the report of workplace assessments 41.To introduce jobs with risk level 1 after identifying above factors
**To identify the hazards of high-standard jobs** 42.To identify the hazards of high-standard jobs (piloting, driving, locomotive driving, firefighting, etc.) based on a review of available resources 43.43.To indigenize the hazards of high-standard jobs (piloting, driving, locomotive driving, firefighting, etc.) based on the assessments performed
**Axis: Controlling occupational hazards**
**To plan for controlling occupational hazards in the occupational health team based on prioritization** 44.To plan for controlling occupational hazards in order of priority after prioritizing the hazardous factors of the workplace
**To control chemical hazards of the workplace** 45.To recommend an alternative chemical in order to control very harmful chemical agents 46.To recommend appropriate engineering methods to control chemical agents in the workplace 47.To offer necessary recommendations to modify the work process to control chemical agents in the workplace 48.To offer appropriate personal protective equipment based on prioritizing hazardous chemicals in the workplace 49.To train all employees how to use personal protective equipment 50.To supervise the correct use of personal protective equipment by employees
**To control the physical hazards of the workplace** 51.To recommend appropriate engineering methods to control the physical exposures of the workplace 52.To offer the necessary recommendations to improve the work process to control the physical exposures of the workplace 53.To offer appropriate personal protective equipment to prioritize hazardous physical exposures in the workplace 54.To train all employees how to use personal protective equipment 55.To supervise the correct use of personal protective equipment by employees
**To control the biological hazards of the workplace** 56.To recommend appropriate engineering methods in order to control biological exposures in the workplace 57.To offer the necessary recommendations to improve the work process to control biological exposures in the workplace 58.To offer appropriate personal protective equipment based on prioritizing biological exposures in the workplace 59.To train all employees how to use personal protective equipment 60.To supervise the correct use of personal protective equipment by employees
**To control the psychological hazards of the workplace** 61.To offer necessary recommendations to improve the work process to control the workplace psychological exposures 62.To train all employees to deal with strategies for coping with stress and factors that disrupt mental health
**To control ergonomic hazards of the workplace** 63.To make workstation modifications based on prioritization in order to control ergonomic exposures in the workplace 64.To offer necessary recommendations to improve the work process to control the workplace ergonomic exposures 65.To train all employees to observe ergonomic principles
**Axis: Determining the appropriate job position for each person (fitness for work)**
**To determine appropriate job position for employees based on demographic characteristics, individual abilities, hazardous factors identified in the workplace, and underlying diseases** 66.To determine the fitness for work of the person for the proposed job based on the results of occupational health assessments 67.To determine the potential risk of employment for employees considering the health status of the individual and his illnesses, the reports of the occupational health team regarding the exposures in the workplace and determining the probability of the impact of these exposures on the individual's health 68.To determine the individual's fitness for work in high-standard occupations (piloting, driving, locomotive driving, firefighting, etc.) according to national and international guidelines 69.To determine the individual’s fitness for work after the necessary assessments when the employer or the supervisor declares that the employee needs to be re-examined 70.To determine the individual’s fitness for work after suffering from an occupational disease 71.To determine how to return to work after suffering from a non-occupational disease 72.To determine how to continue employment after prescribing new medication or special treatment
**To find a person physically fit for the work based on the reports of the occupational health team and the estimated physical workload** 73.To determine the individual’s fitness for work after necessary assessments based on the classification of jobs to very light, light, medium, heavy, and very heavy
**To find a person mentally fit for the work based on the reports of the occupational health team and the estimated mental workload** 74.To determine the individual’s fitness for work after necessary assessments based on the mental workload
**Axis: Occupational health examinations (health risk assessment/medical surveillance)**
**To design occupational health examinations and evaluations in collaboration with other professionals based on the reports of the occupational health team** 75.To design pre-placement (pre-employment) examinations and evaluations for each job according to the reports of the occupational health team and evidence-based practice, considering the imposition of the lowest possible costs 76.To design periodic (screening or surveillance) examinations and evaluations for each job according to the reports of the occupational health team and evidence-based practice, considering the imposition of the lowest possible costs 77.To design exit examinations and evaluations for each job according to the reports of the occupational health team and evidence-based practice, considering the imposition of the lowest possible costs 78.To design occupational health examinations and evaluations for high-standard jobs using an informed decision-making approach based on existing standards and indigenous conditions 79.To select the examinations and evaluations needed to assess mental health based on the job 80.To select the examinations and evaluations needed to assess physical capacity based on the job 81.To select the examinations and evaluations needed to assess functional capacity based on the job
**To conduct occupational health examinations and evaluations based on the designed protocol in collaboration with other professionals** 82.To perform pre-placement examinations for various occupations 83.To perform periodic examinations for various occupations 84.To perform return to work examinations for various occupations 85.To perform fitness for work examinations for various occupations 86.To perform exit examinations for various occupations 87.To perform functional capacity evaluations for various occupations
**To decide about the employment of the individuals based on the assessments** 88.To decide about the start of employment of the individuals based on pre-placement examinations and functional capacity evaluation 89.To decide about the continuation of the employment of the employees based on periodic examinations .90.To decide about the employees' return to work based on a return to work examination 91.To decide about fitness for work of the individuals for a current or proposed job based on the results of fitness for work and functional capacity assessments
**To use all national and international standards and requirements about occupational health services in presenting occupational health services** 92.To apply existing international standards in the provision of occupational health services 93.To apply existing national standards in the provision of occupational health services 94.To identify the best way to provide occupational health services based on existing international and national standards and considering regional, social, and economic conditions
**To report the results of occupational health assessments to the relevant authorities** 95.To observe the professional principles of report writing 96.To observe trustworthiness and honesty in presenting the occupational health assessment reports 97.To observe realism in presenting reports 98.To provide the necessary suggestions to improve the working environment to maintain and improve employees' health 99.To consider the benefits of an employer, employees, and the health system in presenting the report, while respecting the principles of justice
**To provide preventive services for non-occupational diseases to employees** 100.To determine the risk of non-occupational "non-communicable" diseases for employees 101.To provide the necessary recommendations for preventing non-communicable diseases to the employees and their families based on the results of examinations and paraclinical assessments 102.To provide the necessary recommendations for preventing infectious diseases to the employees and their families based on the results of examinations and paraclinical assessments 103.To recommend health promotion methods to employees based on the risk of communicable and non-communicable diseases
**Axis: Managing work-related/occupational diseases**
**To use appropriate methods to diagnose occupational diseases** 104.To choose appropriate diagnostic tests based on scientific evidence and indications and consider the lowest cost and risk for the patient 105.To observe the criteria of usefulness, including availability, non-riskiness, and the lowest possible cost for the patient in choosing diagnostic tests 106.To perform diagnostic tests required for occupational health examinations based on existing guidelines 107.To use appropriate screening methods for early diagnosis of occupational diseases **Respiratory diseases:** 108.To perform lung function tests based on the indication to diagnose occupational respiratory diseases 109.To request appropriate imaging tests based on the indication to diagnose occupational respiratory diseases **Diseases of the hearing system:** 110.To request appropriate audiometric tests based on the indication to diagnose occupational hearing disorders 111.To request appropriate diagnostic tests based on the indication to rule out non-occupational causes of hearing disorders **Musculoskeletal diseases:** 112.To perform appropriate clinical tests based on indication to diagnose musculoskeletal diseases 113.To request appropriate imaging tests based on the indication to diagnose occupational musculoskeletal diseases 114.To request appropriate physiological tests based on the indication to diagnose occupational musculoskeletal diseases **Other occupational diseases:** 115.To perform appropriate clinical tests based on indication to diagnose other occupational diseases 116.To request appropriate imaging tests based on indication to diagnose other occupational diseases 117.To request appropriate paraclinical tests based on the indication to diagnose other occupational diseases
**To use appropriate methods to treat occupational diseases** 118.To recommend to the patient the necessary exercises and movements for the management of musculoskeletal diseases based on the principles of informed decision-making 119.To prescribe the medications needed to manage occupational diseases to the patient based on the principles of informed decision-making
**To apply appropriate methods for rehabilitation and early return to work of employees** 120.To prescribe appropriate rehabilitation methods for the patient according to the type of disease 121.To provide the necessary recommendations for the early return to work of the person 122.To provide the necessary recommendations to improve the workplace environment in case of illness, drug consumption, or special treatment for the employees 123.To recommend to the patient the modifications needed to manage occupational diseases based on the principles of informed decision-making
**To identify the relationship between diseases and hazardous factors in the workplace** 124.To determine the relationship between the diagnosed disease and hazardous factors in the workplace based on scientific evidence 125.To determine the impact of the job on the diagnosed disease based on scientific evidence 126.To apportion the contribution of occupational factors on the diagnosed disease based on scientific evidence 127.To determine the impact of non-occupational factors on the diagnosed disease based on scientific evidence
**To register and report occupational diseases based on national and international guidelines** 128.To register the disease diagnosed as occupational in the relevant sites based on national/international guidelines 129.To report specific occupational diseases to the competent authorities based on national/international guidelines
**Axis: Inter-organizational and inter-disciplinary relations and legal judgment**
**To cooperate with external organizations (forensic medicine, insurance organizations, medical council, judiciary, and labor office)** 130.To request information related to workplace assessments and occupational health examinations to prepare responses to consultations of institutions and organizations 131.To comment on the relationship between the disease and the job, based on the information provided by the occupational health team and others when requested by external organizations 132.To apportion the contribution of occupational factors in causing disease based on the information provided by the occupational health team and other investigations when external organizations request 133.To determine the degree of the employee's impairment based on the existing standards when requested by relevant institutions or organizations 134.To determine the degree of employee’s disability based on the relevant existing protocols 135.To determine the extent of the employer's negligence in causing the employee's disease, disability, or death, in cooperation with the relevant team members, based on the information provided by the occupational health team and other investigations 136.To determine the extent of negligence of the members of the occupational health team in causing an employee's disease, disability, or death in cooperation with the relevant team members, based on the information provided by the occupational health team and other investigations 137.To give technical advice to industrial owners and employers regarding improving the workplace, preventing occupational diseases, and early identification of occupational diseases, if requested 138.To respond effectively to external and internal organizations' consultations in written and oral form
**To seek the consultation of specialists from other disciplines only when necessary for the proper diagnosis and treatment of occupational and non-occupational diseases and respond to their consultation** 139.To seek consultation from specialists in other fields for properly rehabilitating patients with occupational diseases 140.To refer the patient to specialists of other disciplines to complete the diagnosis process and rule out non-occupational causes of diseases when necessary 141.To refer the person to specialists of other disciplines to complete the treatment process when necessary 142.To refer the person to specialists of other disciplines to complete the rehabilitation process when necessary 143.To seek consultation to rule out non-occupational causes of diseases 144.To respond to the consultations of general practitioners and specialists from other disciplines in the field of occupational diseases 145.To give consultation to other members of the occupational health team in their field of expertise 146.To use the principles of personal and professional development to improve his (her) performance and other members of the occupational health team 147.To cooperate in inter-organizational teams to determine hazard and hazardous jobs 148.To provide effective feedback to the members of the occupational health team in the interprofessional team
**To cooperate in monitoring the provision of occupational health services** 149.To cooperate in monitoring the centers of providing occupational hygiene services based on national and international standards 150.To cooperate in monitoring the centers of providing occupational medicine services based on national and international standards 151.To cooperate in monitoring high-standard jobs based on national and international standards 152.To perform economic evaluation and prioritization of programs to manage existing and organizational resources
**Axis: Education and scholarship in providing occupational health services**
**To carry out professional activities based on a scholarly approach** 153.To find the required evidence in valid databases using appropriate search strategies 154.To retrieve the searched evidence using appropriate and valid criteria 155.To critically evaluate searched evidence using valid criteria and to be able to apply relevant results 156.To participate in research activities in the field of occupational health in interprofessional teams 157.To design appropriate research to solve occupational health problems (problem-oriented research) 158.To use the evidence and results of domestic research in professional performance and promotion of occupational health services 159.To make decisions by evaluating the available evidence and considering the background factors and expert opinions 160.To compile instructions/guidelines related to occupational health with emphasis on domestic characteristics based on the specialized opinions of the members of the occupational health team
**To collaborate in educating different stakeholders** 161.To conduct needs assessment regarding the required training for different stakeholders 162.To design training suitable for different audiences (employees, employers, and managers in occupational health) 163.To carry out training suitable for different audiences (employees, employers, and managers in the occupational health field) 164.To evaluate training suitable for different audiences (employees, employers, and managers in the occupational health field)

In the task-based learning strategy, it is required to determine general and special competencies to conduct each task. The matrix of tasks- general and specialized competencies in two axes (as a sample) is shown in Table 3.

**Table 3:** Matrix of Task-Competency (general and specialized) in an interprofessional training program based on a task-based approach

Tasks	Specialized competency								General competency			
	**Identification of occupational exposures**	**Mastery in exposure measurement**	**Interpretation of results**	**Medical history taking**	**Occupational history taking**	**Physical exam**	**Professional judgment**	**Mastery in the methods of exposure control**	**Communication skills**	**Interprofessional collaboration**	**Professionalism**	**Evidence-based decision making**
**Axis: Identifying and assessing the risk of workplace hazards (exposure risk assessment)**												
To carry out an initial assessment of the work environment in the form of an occupational health team (walkthrough survey)	**+**	**+**	**+**	**-**	**±**	**-**	**±**	**-**	**+**	**+**	**+**	**+**
To design a plan for quantitative environmental exposure measurements based on the initial assessment results	**+**	**+**	**+**	**-**	**-**	**-**	**-**	**-**	**-**	±	**+**	+
To design a plan for personal quantitative exposure measurements based on the initial assessment results	**+**	**+**	±	**-**	**-**	**-**	**-**	**-**	**+**	**+**	**+**	**+**
To measure the psychological hazards of the workplace	**+**	**+**	±	**-**	**+**	**-**	**-**	**-**	**+**	**+**	**+**	**+**
To measure ergonomic hazards of the workplace	**+**	**+**	**+**	**-**	**+**	**-**	**+**	**-**	**+**	**+**	**+**	**+**
To compile an occupational exposure matrix in the occupational health team based on the measurements	+	±	+	-	-	-	+	-	±	+	+	+
To prepare a report of assessments and measurements of workplace exposures in cooperation with the members of the occupational health team	+	±	+	-	±	-	+	±	+	+	+	+
To interpret the results of the measurements and present them to different stakeholders	+	±	+	-	±	-	+	±	+	+	+	+
To identify occupations with risk level 1	**+**	**-**	±	**+**	**+**	**-**	**+**	**-**	**+**	**+**	**+**	**+**
To identify the hazards of high-standard jobs	**+**	±	**+**	**-**	**-**	**-**	±	**-**	±	**+**	**+**	**+**
**Axis: Controlling occupational hazards**												
To plan for controlling occupational hazards in the occupational health team based on prioritization	**+**	**-**	**+**	**-**	**-**	**-**	±	**+**	**+**	**+**	**+**	**+**
To control chemical hazards of the workplace	+	-	+	-	-	-	+	+	+	+	+	+
To control physical hazards of the workplace	+	-	+	-	-	-	+	+	+	+	+	+
To control biological hazards of the workplace	+	-	+	-	-	-	+	+	+	+	+	+
To control psychological hazards of the workplace	+	-	+	-	-	-	+	+	+	+	+	+
To control ergonomic hazards of the workplace	+	-	+	-	-	-	+	+	+	+	+	+
**Axis: Determining the appropriate job position for each person (fitness for work)**												
To determine appropriate job positions for employees based on demographic characteristics, individual abilities, hazardous factors identified in the workplace, and underlying diseases	+	-	+	+	+	±	+	-	+	+	+	+
To find a person physically fit for the work based on the reports of the occupational health team and the estimated physical workload	+	-	+	+	-	+	+	-	+	+	+	+
To find a person mentally fit for the work based on the reports of the occupational health team and the estimated mental workload	+	-	+	+	-	+	+	-	+	+	+	+
**Axis: Occupational health examinations (health risk assessment)**												
To design occupational health examinations and evaluations in collaboration with other professionals based on the reports of the occupational health team	+	-	+	-	+	-	+	-	+	+	+	+
To conduct occupational health examinations and evaluations based on the designed protocol in collaboration with other professionals	±	-	-	+	+	+	-	-	+	+	+	-
To decide about the employment of the individuals based on the assessments	+	-	±	-	-	-	+	-	+	+	+	+
To use all national and international standards and requirements about occupational health services in presenting occupational health services	-	-	-	-	-	-	-	-	+	+	+	+
To report the results of occupational health assessments to the relevant authorities	-	-	-	-	-	-	-	-	+	+	+	+
To provide preventive services for non-occupational diseases to employees	-	-	±	+	+	+	+	-	+	+	+	+

	**Specialized competencies**												**General competencies**			
**Tasks**	**Identification of occupational exposures**	**Interpretation of the results of measurements**	**Medical history taking**	**Occupational history taking**	**Physical exam**	**Selecting appropriate paraclinical tests**	**Interpretation of paraclinical tests**	**Diagnosis of disease**	**Diagnosis of disease as work-induced**	**Management of disease treatment**	**Management of disease rehabilitation**	**Professional judgment**	**Communication skills**	**Interprofessional collaboration**	**Professionalism**	**Evidence-based decision making**
**Axis: Managing work-related/occupational diseases**																
To use appropriate methods to diagnose occupational diseases	-	-	+	+	+	+	+	+	+	±	±	+	+	+	+	+
To use appropriate methods to treat occupational diseases	-	-	+	+	+	+	+	+	±	**+**	±	+	+	+	+	+
To apply appropriate methods for rehabilitation and early return to work of employees	±	±	+	+	+	+	+	+	±	±	+	+	+	+	+	+
To identify the relationship between diseases and hazardous factors in the workplace	+	+	+	+	-	-	-	+	+	-	-	+	+	+	+	+
To register and report occupational diseases based on national and international guidelines	-	-	-	-	-	-	-	-	-	-	-	±	+	+	+	+

Tasks	**Specialized skill**				**General skill**			
	Mastery of national and international guidelines/instructions	Mastery in research methods	Professional judgment	Mastery in education methods	Communication skills	Interprofessional collaboration	Professionalism	Evidence-based decision making
To carry out professional activities based on a scholarly approach	+	+	+	-	+	+	+	+
To collaborate in educating different stakeholders	-	-	-	-	+	+	+	+

*Applies + , may apply ± , does not apply -

In planning a task-based learning program, it is often helpful to prepare a matrix on which the specified tasks are related to the roles. The tasks-roles matrix of the members of the occupational health team is shown in Table 4.

**Table 4:** the Tasks- roles matrix of the members of the occupational health team

Tasks		Roles							
		Therapist	Consultant	Educator	evaluator	Screener	Professional/expert	Researcher	Monitor
**Axis: Identifying and assessing the risk of workplace hazards (exposure risk assessment)**									
To carry out an initial assessment of the work environment in the form of an occupational health team (walkthrough survey)		**-**	** + **	** ± **	** + **	** ± **	** + **	** ± **	** ± **
To design a plan for quantitative environmental exposure measurements based on the initial assessment results		**-**	** ± **	** + **	** + **	** ± **	** ± **	** ± **	**-**
To design a plan for personal quantitative exposure measurements based on the initial assessment results		**-**	** ± **	** + **	** + **	** ± **	** ± **	** ± **	**-**
To measure the psychological hazards of the workplace		**-**	** ± **	** + **	** + **	** ± **	** ± **	** ± **	**-**
To measure ergonomic hazards of the workplace		**-**	** ± **	** + **	** + **	** ± **	** ± **	** ± **	**-**
To compile an occupational exposure matrix in the occupational health team based on the measurements		**-**	** ± **	** ± **	** + **	** ± **	** + **	**-**	**-**
To prepare a report of assessments and measurements of workplace exposures in cooperation with the members of the occupational health team		**-**	** ± **	** ± **	** + **	** ± **	** + **	**-**	**-**
To interpret the results of the measurements and present them to different stakeholders		**-**	** ± **	** ± **	** + **	** ± **	** + **	**-**	**-**
To identify occupations with risk level 1		** ± **	** + **	** + **	** + **	** + **	** + **	** + **	** ± **
To identify the hazards of high-standard jobs		**-**	** + **	** + **	** + **	** ± **	** + **	** ± **	**-**
**Axis: Controlling occupational hazards**									
To plan for controlling occupational hazards in the occupational health team based on prioritization		**-**	** + **	** + **	** + **	** + **	** + **	**-**	**-**
To control chemical hazards of the workplace		**-**	** + **	** ± **	** + **	**-**	** ± **	**-**	**-**
To control physical hazards of the workplace		**-**	** + **	** ± **	** + **	**-**	** ± **	**-**	**-**
To control biological hazards of the workplace		**-**	** + **	** ± **	** + **	**-**	** ± **	**-**	**-**
To control psychological hazards of the workplace		**-**	** + **	** ± **	** + **	**-**	** ± **	**-**	**-**
To control ergonomic hazards of the workplace		**-**	** + **	** ± **	** + **	**-**	** ± **	**-**	**-**
**Axis: Determining the appropriate job position for each person (fitness for work)**									
To determine appropriate job positions for employees based on demographic characteristics, individual abilities, hazardous factors identified in the workplace, and underlying diseases		-	+	±	±	+	+	-	-
To find a person physically fit for the work based on the reports of the occupational health team and the estimated physical workload		-	±	±	+	+	+	-	-
To find a person mentally fit for the work based on the reports of the occupational health team and the estimated mental workload		-	±	±	+	+	+	-	-
**Axis: Occupational health examinations (health risk assessment)**									
To design occupational health examinations and evaluations in collaboration with other professionals based on the reports of the occupational health team	-		+	±	+	+	+	-	-
To conduct occupational health examinations and evaluations based on the designed protocol in collaboration with other professionals	-		-	-	±	+	+	-	-
To decide about the employment of the individuals based on the assessments	-		+	-	±	±	+	-	-
To use all national and international standards and requirements about occupational health services in presenting occupational health services	±		-	-	+	+	+	-	+
To report the results of occupational health assessments to the relevant authorities	±		-	-	+	+	+	-	+
To provide preventive services for non-occupational diseases to employees	+		** + **	** ± **	**-**	** + **	** + **	**-**	**-**
**Axis: Managing work-related/occupational diseases**									
To use appropriate methods to diagnose occupational diseases	** + **		** ± **	**-**	**-**	** ± **	** + **	**-**	**-**
To use appropriate methods to treat occupational diseases	** + **		** ± **	**-**	**-**	** ± **	** + **	**-**	**-**
To apply appropriate methods for rehabilitation and early return to work of employees	+		±	-	-	±	+	-	-
To identify the relationship between diseases and hazardous factors in the workplace	±		±	-	±	±	+	-	-
To register and report occupational diseases based on national and international guidelines	+		-	-	-	+	+	-	-
**Axis: Inter-organizational and inter-disciplinary relations and legal judgment**									
To cooperate with external organizations (forensic medicine, insurance organizations, medical council, judiciary, and labor office)	+		+	-	+	+	+	±	-
To seek the consultation of specialists from other disciplines only when necessary for the proper diagnosis and treatment of occupational and non-occupational diseases and respond to their consultations	+		-	-	-	±	+	-	-
To cooperate in monitoring the provision of occupational health services	-		-	-	±	+	+	-	+
**Axis: Education and scholarship in providing occupational health services**									
To carry out professional activities based on a scholarly approach	+		+	+	+	+	+	+	+
To collaborate in educating different stakeholders	**-**		**-**	** + **	**-**	**-**	** + **	** + **	**-**

*Applies + , may apply ± , does not apply -

In the final step of program development, teaching and learning methods in the interprofessional situation and assessment methods were determined based on each task. The matrix of teaching–learning and assessment methods in the interprofessional task based-learning program is shown in Table 5.

**Table 5:** matrix of tasks and methods of teaching–learning and methods of assessment of learners in the interprofessional task based learning program

Tasks	Teaching–learning method					Assessment method				
	**Interprofessional Problem-solving based method**	**Interprofessional small groups**	**Interprofessional Simulation**	**Interprofessional Workplace-based**	**Interprofessional Blended/virtual learning**	**Written**	**Reasoning**	**Simulation**	**Observational**	**Performance recording**
**Axis: Identifying and assessing the risk of workplace hazards (exposure risk assessment)**										
To carry out an initial assessment of the work environment in the form of an occupational health team (walkthrough survey)	Team project	Interprofessional discussion (based on working round)		Interprofessional working round		Project/report				Logbook/Portfolio
To design a plan for quantitative environmental exposure measurements based on the initial assessment results			Teaching in a simulated environment	Interprofessional project in the real environment		Designing written project		Simulation/Project-based	Observational/short case	Logbook/Portfolio
To design a plan for personal quantitative exposure measurements based on the results of the initial assessment			Teaching in a simulated environment	Interprofessional project in the real environment		Designing written project/ Project-based			Observational /short case	Logbook/Portfolio
To measure the psychological hazards of the workplace	Scenario/case-based teaching				Virtual teaching (revers)	Simulation/Project-based	P.M.P/KF			Logbook/Portfolio
To measure ergonomic hazards of the workplace	Scenario/case-based teaching/Problem solving based	Small group discussion	Discussion based on film and virtual reality		Virtual teaching (revers)	Project-based	P.M.P/KF	Simulation-based (written)	Observational/practical	Logbook/Portfolio
To compile an occupational exposure matrix in the occupational health team based on the measurements	Problem-solving based	Concept map				Modified written/scenario-based	P.M.P-KF	Station-based simulation		Logbook/Portfolio
To prepare a report of assessments and measurements of workplace exposures in cooperation with the members of the occupational health team	Project-based learning	Interprofessional group Discussion			E-learning and Filliped classroom	Project-based assessment				Logbook/Portfolio
To interpret the results of the measurements and present them to different stakeholders	simulation/case-based learning	Interprofessional small group			e-learning	Essay-Modified Essay	P.M.P-KF			Logbook/Portfolio
To identify occupations with risk level 1	Project-based learning in an interprofessional team	Interprofessional small group		Interprofessional rounds		Modified essay examination	P.M.P-KF			Logbook/ Portfolio
To identify the hazards of high-standard jobs	Project-based learning in an interprofessional team	Interprofessional small group		Interprofessional rounds			P.M.P-KF			Logbook/ Portfolio
**Axis: Controlling occupational hazards**										
To plan for controlling occupational hazards in the occupational health team based on prioritization	simulation/case-based learning	Interprofessional small group				Modified essay examination	P.M.P-KF		Case-based Discussion	Logbook/Portfolio
To control chemical hazards of the workplace	simulation/case-based learning	Interprofessional small group				Modified essay examination	P.M.P-KF		Case-based Discussion	Logbook/ Portfolio
To control physical hazards of the workplace	simulation/case-based learning	Interprofessional small group				Modified essay examination	P.M.P-KF		Case-based Discussion	Logbook/ Portfolio
To control biological hazards of the workplace	simulation/case-based learning	Interprofessional small group				Modified essay examination	P.M.P-KF		Case-based Discussion	Logbook/ Portfolio
To control psychological hazards of the workplace	simulation/case-based learning	Interprofessional small group				Modified essay examination	P.M.P-KF		Case-based Discussion	Logbook/ Portfolio
To control ergonomic hazards of the workplace	simulation/case-based learning	Interprofessional small group	film and Interprofessional discussion		e-learning	Modified essay examination Modified essay examination	P.M.P-KF		Case-based Discussion	Logbook/ Portfolio
**Axis: Determining the appropriate job position for each person (fitness for work)**										
To determine appropriate job positions for employees based on demographic characteristics, individual abilities, hazardous factors identified in the workplace, and underlying diseases	Case-based learning/scenario-reasoning teaching methods	Interprofessional small group	Simulation	Ambulatory care learning		Modified essay examination	P.M.P-KF		Case-based Discussion Observational Examination (MINI-CEX)	Logbook/Portfolio
To find a person physically fit for the work based on the reports of the occupational health team and the estimated physical workload	Case-based learning/scenario-reasoning teaching methods	Interprofessional small group	Simulation	Ambulatory care learning		Modified essay examination	P.M.P-KF		Case-based Discussion Observational Examination (MINI-CEX)	Logbook/Portfolio
To find a person mentally fit for the work based on the reports of the occupational health team and the estimated mental workload	Case-based learning/scenario-reasoning teaching methods	Interprofessional small group	Simulation	Ambulatory care learning		Modified essay examination	P.M.P-KF		Observational Examination (MINI-CEX)	Logbook/Portfolio
**Axis: Occupational health examinations (health risk assessment)**										
To design occupational health examinations and evaluations in collaboration with other professionals based on the reports of the occupational health team	Case-based learning/scenario-reasoning teaching methods	Interprofessional small group	Simulation			Modified essay examination	P.M.P-KF		Case-based Discussion	Logbook/Portfolio
To conduct occupational health examinations and evaluations based on the designed protocol in collaboration with other professionals		Peer learning	simulation: role play/ROLE MODEL Simulated patient	Ambulatory care learning				OSCE Simulation Examination	MINI-CEX	Logbook/Portfolio
To decide about the employment of the individuals based on the assessments	Case-based learning/scenario-reasoning teaching methods	Interprofessional small group	Simulation	Ambulatory care learning		Modified essay examination	P.M.P-KF		Case-based Discussion	Logbook/Portfolio
To use all national and international standards and requirements about occupational health services in presenting occupational health services	Case-based learning/scenario-reasoning teaching methods				E-learning, Flipped classroom	Modified essay examination				Logbook/Portfolio
To report the results of occupational health assessments to the relevant authorities	Case-based learning/scenario-reasoning teaching methods Project-based learning				E-learning, Flipped classroom	Modified essay examination				Logbook/Portfolio
To provide preventive services for non-occupational diseases to employees	Case-based learning/scenario-reasoning teaching methods	Interprofessional small group			E-learning, Flipped classroom	Modified essay examination	P.M.P-KF		Case-based Discussion	Logbook/Portfolio
**Axis: Managing work-related/occupational diseases**										
To use appropriate methods to diagnose occupational diseases	Case-based learning/scenario-reasoning teaching methods		Simulation	Ambulatory care learning		Modified essay examination	P.M.P-KF		Case-based Discussion Observational Examination (- DOPS. MINI-CEX)	Logbook/ Portfolio
To use appropriate methods to treat occupational diseases	Case-based learning/scenario-reasoning teaching methods		Simulation	Ambulatory care learning		Modified essay examination	P.M.P-KF		Case-based Discussion Observational Examination (MINI-CEX)	Logbook/ Portfolio
To apply appropriate methods for rehabilitation and early return to work of employees	Case-based learning/scenario-reasoning teaching methods		Simulation	Ambulatory care learning		Modified essay examination	P.M.P-KF		Case-based Discussion Observational Examination (-MINI-CEX)	Logbook/Portfolio
To identify the relationship between diseases and hazardous factors in the workplace	Case-based learning/scenario-reasoning teaching methods	Interprofessional small group			E-learning, Flipped classroom	Modified essay examination	P.M.P-KF		Case-based Discussion	Logbook/Portfolio
To register and report occupational diseases based on national and international guidelines	Case-based learning/scenario-reasoning teaching methods		Simulation		E-learning, Flipped classroom	Essay				Logbook/Portfolio
**Axis: Inter-organizational and inter-disciplinary relations and legal judgment**										
To cooperate with external organizations (forensic medicine, insurance organizations, medical council, judiciary, and labor office)	Case-based learning/scenario-reasoning teaching methods	Interprofessional small group	Role play- Simulation	Workplace-based education		Modified essay examination	P.M.P-KF	Objective structured examination TOSCE	Case-based Discussion Observational Examination (MINI-CEX)	Logbook/Portfolio
To seek the consultation of specialists from other disciplines only when necessary for the proper diagnosis and treatment of occupational and non-occupational diseases and respond to their consultations	Case-based learning/scenario-reasoning teaching methods	Interprofessional small group	Role-play Simulation	Workplace-based education		Modified essay examination	P.M.P-KF	TOSCE- Objective structured examination	Case-based Discussion Observational Examination (MINI-CEX)	Logbook/Portfolio
To cooperate in monitoring the provision of occupational health services	Case-based learning/scenario-reasoning teaching methods			Workplace-based education	E-learning, Flipped classroom	Modified essay examination			Case-based Discussion	Logbook/Portfolio
**Axis: Education and scholarship in providing occupational health services**										
To carry out professional activities based on a scholarly approach	Case-based learning/scenario-reasoning teaching methods Project-based learning				E-learning, Flipped classroom	Project-based assessment Modified essay examination				Logbook/Portfolio
To collaborate in educating different stakeholders	Case-based learning/scenario-reasoning teaching methods	Interprofessional Small Group	Simulation Role play/ -Role Model					Observation Examination /Objective structured examination/TOSCE		Logbook/Portfolio

## Discussion

The task-based strategy provides situations for students to learn the tasks that correspond to their future job duties and acquire the necessary competencies. In this study, a task-based interprofessional training program was developed for different learners of the occupational health team. Different groups are involved in providing occupational health services, among which occupational medicine and occupational hygiene specialists have the most important role in providing the services in our country.

The expected duties of people involved in providing occupational health services need to be reformed proportionately in response to changes in the work environment, technological developments, production processes and the emergence of new hazards in the work environment, and changes in the framework of occupational health laws and guidelines [2, 16, 35–40]. Several studies have been conducted to explain the competencies, skills, and duties of occupational health team members worldwide [2, 16, 35–40]. Their results can be used for reforming and modifying existing curricula and improving the level of occupational health services [41]. In the present study, the integration of interprofessional education and task-based education strategies was used. Interprofessional education provides opportunities for learners to learn technical skills (identifying and assessing the risk of workplace hazards, controlling occupational hazards, determining the appropriate job position, occupational health examinations, and managing work-related/occupational diseases) and soft and non-technical skills (interprofessional collaboration, communication, teamwork, and professionalism) through, from, with and about each other to improve collaboration and the quality of services. The interprofessional competencies as a general competency including role and responsibility, values and professionalism, interprofessional communication, teamwork, and collaboration were considered in different extracted areas.

In the present study, the interprofessional competencies which were introduced by the Interprofessional Education Collaborative Report (IPEC) were used [28]. A planned educational process provides interprofessional situations, where learners learn the tasks expected in their future careers in the real environment and with colleagues from different disciplines with whom they will work in the future [42].

Occupational health is a multidisciplinary, interprofessional, inter-departmental, and inter-organizational field that goes beyond the boundaries of the health sector [43]. Even in the health sector, many people are influential in providing and promoting occupational health, including general physicians, specialists in occupational medicine, and professionals in occupational hygiene, safety, ergonomics, environmental health, etc. Therefore, to ensure the highest level of occupational health for workers and their families, the cooperation of different people in these fields is needed, along with the cooperation of employers, insurance organizations, and other institutions [15].

Interprofessional collaboration is sharing responsibilities while defining the roles and goals of each profession, and integration and shared identities are less important than collaboration as teamwork [44]. Integrating the inter-professional or task-based method that takes advantage of the essential elements of problem-based education and education in small groups can provide an excellent opportunity to understand the course material, teach the material more effectively, and increase the efficiency of the teaching–learning process. Although traditional teaching methods such as lectures are still used in many educational institutions in our country as the primary teaching method, task-based teaching has been implemented in many studies, and its effect on people's learning has been proven [23, 45, 46].

Changing the teaching methods and moving towards more practical training is always challenging at first and faces resistance. In a study in Pakistan, Ayub Khan and his colleagues implemented training on how to prevent surgical wound infection based on interprofessional task-based training, and the results of the study showed a positive effect on knowledge and performance in addition to student satisfaction [23]. In our program, the teaching–learning and assessment methods were designed in real and simulated interprofessional situations. The teaching–learning methods were designed to provide simulated situations and real environments for learners to learn their competencies. The interprofessional small group setting, simulation (case-based learning, scenario, and reasoning teaching methods), and workplace-based learning method (ambulatory-based learning, interprofessional rounds, and project-based learning) were defined as the primary methods. In addition, learner assessment methods were designed to assess the competencies in different domains such as knowledge, skills, and attitudes by different assessment methods in simulated and real situations. The assessment methods included modified essays, team objective structured examinations, and observational examinations, such as mini-clinical exams (Mini-CEX), and direct observation of procedural skills (DOPS). The learners must show their competencies to conduct the expected tasks in their future careers.

In the current study, the tasks of the members of the occupational health team were summarized in seven axes with emphasis on two fields of occupational hygiene and occupational medicine, including: "identifying and assessing the risk of workplace hazards", "controlling occupational hazards", "determining the appropriate job position for each person", "occupational health examinations", "managing work-related/occupational diseases", "inter-organizational and inter-disciplinary relations and legal judgment", and "education and scholarship in providing occupational health services. "

The "Identifying and assessing the risk of workplace hazards" axis is known as one of the most fundamental tasks of the occupational health team. In this task, members of occupational health team, especially occupational hygiene specialists, identify and assess all types of hazards and exposures in the workplace based on existing standards. The provision of any occupational health service depends on the correct and complete performance of this task and appropriate reporting of its results. The diversity of job exposures in different environments shows the complexity of this task and the need to have skills to perform it [47]. Incomplete or incorrect information in this regard can lead to incorrect decisions in all areas of occupational health, including selecting a suitable person for a job, diagnosing a disease as occupational, and even legal decisions. Therefore, interprofessional team members must acquire the necessary skills such as methods of measurement, interpretation, and reporting of the assessments to perform this task in different situations. In this task, students must evaluate hazards, including chemical, physical, ergonomic, biological, and psychological exposures in the real environment of the workplace. Based on the initial assessment results, the learners should formulate a plan for individual and environmental quantitative measurements and compile a matrix of occupational exposures of the employees. In the next step, the learners should compile, interpret, and report the results of assessments and measurements of the hazardous factors of the work environment in cooperation with the members of the occupational health team.

The axis of "controlling occupational hazards" deals with the standardization of exposure to hazardous factors in the workplace. The basis of this task is to reduce the hazards caused by occupational exposures. Therefore, especially for perilous hazards such as ionizing radiation or carcinogenic agents, the goal is to eliminate the exposure or control it as much as possible. For other exposures, when there is an exposure that exceeds the permissible limits based on the report of exposures in the work environment (previous task), it is necessary to put exposure control on the agenda. There are various methods to control exposures, from replacing a highly hazardous exposure with a less hazardous exposure as the highest level of exposure control to reducing the concentration or intensity of exposure using measures such as installing ventilation, and finally using personal protective equipment [48]. Planning to prioritize to control chemical, physical, biological, psychological, and ergonomic hazards are explained in this task. It is expected that members of occupational health team can play an influential role in reducing the burden of occupational diseases, increasing job satisfaction, and increasing productivity with interprofessional cooperation. In ​​hazard control, the control of hazardous chemical, physical, and psychological factors was emphasized in this study, and control of chemical and physical hazards was considered a task exclusively performed by occupational hygiene professionals, and it is less commonly considered as a team task. Determining, managing, and controlling psychological hazards in the work environment is another task emphasized in this axis. The importance of this category in work environments is that it has a significant effect on job satisfaction, reducing the mental burden of the work and increasing productivity; the educational curricula of the disciplines related to occupational health have not paid enough attention to this task regarding psychological hazards, therefore, in practice, occupational health teams do not pay much attention to this critical occupational hazard in the assessments of the work environment.

One of the essential tasks of the occupational health team is to determine the appropriate job position for each person, taking into account physical, mental, and social limitations and abilities. In this task, after identifying the occupational tasks of the person, determining the exposures, evaluating the individual’s physical and mental health status, and evaluation of his (her) functional capacity, it is necessary to put these categories together to select the right person for the job. Accordingly, in this task, the occupational health team must decide whether the job applicant can perform the main tasks of the job efficiently or not and what changes are needed in the workplace so that the person can work more efficiently. This task is very sensitive because a mistake in a decision can lead to aggravating or inducing an occupational disease or, on the contrary, prevent a person from employment in a job that he (she) is capable of doing it. In this task, the following decisions may be made for the job applicant: "fit for the job", "fit subject to changes in the workplace", "fit with occupational restrictions/limitations", and "unfit for the job" [49]. Due to the variety of jobs and significant differences between work environments, even in similar industries, there is no reliable standard in this field [49], so this task is performed based on available reports and individual assessments. In this task, the members of the occupational health team must work together, based on the estimation of the burden of occupational hazards and hazardous factors identified in the workplace and underlying diseases, to determine the appropriate job position for the employees.

Another focus of the tasks of the occupational health team, especially general practitioners and occupational medicine specialists, is "occupational health examinations." In this task, occupational medicine specialists design various types of occupational health examinations based on the reports of the occupational health team. These examinations include "pre-employment (pre-placement)", "periodic (screening)", "return to work", "fitness for work", and "exit" examinations. In most countries, entry into any job requires an initial examination of the applicant based on workplace exposures, which are carried out as pre-employment or pre-placement examinations. People working in different workplaces need to be examined periodically to detect the possible effect of occupational exposures on the employee’s health, i.e., early case finding, which is done in the form of periodic examinations based on the guidelines in each country [50]. Any person who is away from work for a period due to a serious illness must be re-evaluated in terms of fitness for work when returning to work, which is done in the form of return-to-work examinations. In addition, according to the employer's report, any employed person who cannot perform the assigned tasks must be re-evaluated in terms of fitness for work. Another time a person needs to be checked is when he (she) leaves any work environment for any reason (dismissal, retirement, job change, etc.). In most of the examinations that are performed, in addition to determining the level of health, the condition of fitness of the individual for work is also evaluated [51]. In this task, the learners of occupational health must design and implement occupational examinations and make decisions based on the results and related national and international standards and requirements. Reporting the results of the examinations to different stakeholders based on different perspectives is also performed in this task.

In the current study, "managing occupational/work-related diseases", focuses on diagnosing, treating, and rehabilitating work-related diseases. Occupational health services play an essential role in preventing, treating, and rehabilitating work-related diseases [52]. To reach the strategy of the World Health Organization, "occupational health for all", it is necessary to provide effective occupational health services to all employees by competent people in this field [53]. This requires empowerment and proper training based on the professional needs and practical tasks of the individuals involved in providing occupational health services. Considering the importance of health care in people's familial, social and economic life, evaluation and monitoring of the efficiency and effectiveness of occupational health services are performed by many people and organizations involved in this field and outside the occupational health team, such as insurance companies, employers, health departments of universities of medical sciences, etc. In this task, learners of different professions ​​are expected to actively cooperate in diagnosing occupational diseases. They suggest suitable methods for preventing and treating occupational diseases, appropriate rehabilitation, and early or progressive return to work. Identifying the relationship between disease and hazardous factors in the work environment is also essential. The results must be recorded and reported by occupational health team based on national and international guidelines. One of the neglected tasks in providing occupational health services is using proper rehabilitation methods for rehabilitation and early return to work of employees. In this category, the authority of the occupational health team is somewhat limited, and many factors are involved in this decision. The factors include the certificate of the treating physicians for when and how to return to work, the policies of insurance companies, the policies of the workplace, leave benefits, etc.

One of the duties of the members of occupational health team, especially occupational medicine specialists, is defined in the axis of "inter-organizational, interdisciplinary relations and legal judgment." One of the world's most essential costs of occupational health is the cost of occupational compensation due to occupational diseases or accidents. In this axis, the tasks of the occupational health team include determining whether the disease is occupational, determining the percentage of occupational involvement in causing the disease (apportionment), and determining impairment and disability [54], which is performed in the form of consulting relevant institutions, including forensic medicine organization, labor office, insurance companies, health departments of universities, etc. In this task, consultation with external organizations and various stakeholders and monitoring the provision of occupational health is essential. Involvement in intra and extra-organizational activities, such as cooperation with forensic medicine organization, Health system, judiciary, and labor office related to the provision of occupational health services is explained in this axis. Neglecting interprofessional and inter-departmental cooperation for health team members can be due to the lack of a comprehensive view of the activities of the occupational health team. This can be improved by using the strategy of interprofessional education and the development of professional commitment competencies.

Finally, the axis of "education and scholarship in the provision of occupational health services" emphasizes the two primary tasks of teaching different stakeholders and using the scholarly strategy in professional activities. The occupational health team needs to train different stakeholders such as employees, employers, colleagues, etc.; therefore, it is necessary to design, implement and evaluate the appropriate training process. In this axis, a scholarly strategy for all professional activities has been considered.

In this study a task-competency matrix was compiled in line with a competency-based framework of ACGME (Accreditation Council for Graduate Medical Education) [55]. In this matrix, the principal competencies to perform each task was defined. In each axis two kinds of competencies, including general competencies (communication skills, interprofessional collaboration, professionalism, and evidence-based decision making), and specialized competencies (identification of occupational exposures, mastery in exposure measurement, interpretation of results, medical history taking, occupational history taking, physical exam, professional judgment, and mastery in the methods of exposure control) were emphasized and scored according to the importance and applicability of each competency to perform each task, for example “professional judgment” as a competency was important in “determining appropriate job position” task, less important in “walkthrough survey”, and not important in “measuring the psychological hazards of the workplace”. This classification was done for the required competencies in each axis.

In this regard, “interpretation of results”, “identification of occupational exposures”, “professional judgment”, and “mastery in the methods of exposure control” were emphasized as the most important specialized competencies in “controlling occupational hazards” axis. In the second axis, i.e. “fitness for work”, interpretation of the initial assessments and judgment to find the appropriate job for a person or find a person completely fit for performing essential tasks of the job is very important, therefore, the following competencies were rated important: “interpretation of results”, “occupational history taking”, “medical history taking”, “professional judgment”, “physical exam”, and “identification of occupational exposures”. In the axis of “health risk assessment”, two specialized competencies, i.e. “occupational history taking”, and “professional judgment” were considered as the most important competencies. This axis is performed mostly by occupational physicians, so there was not a competency with high degree of necessity for all tasks. The forth axis was “managing work-related/occupational diseases”, an important task which is again performed by physicians, especially occupational medicine specialists; in this axis, the physician should diagnose, treat, rehabilitate, and most importantly prevent occupational diseases, so the following tasks which are mostly medical tasks were scored as the most important: “medical history taking”, “occupational history taking”, “physical exam”, “selecting appropriate paraclinical tests”, “interpretation of paraclinical tests”, “diagnosis of disease”, and “diagnosis of disease as work-induced”.

The results showed that general competencies such as “communication skills”, “evidence-based decision making”, “interprofessional collaboration’, and “professionalism” had an effective role in all tasks. Performing tasks related to occupational health services there is a need for collaboration of the members of different professions such as occupational medicine, industrial hygiene, and ergonomics. In addition, due to the emergence of new exposures and hazards in the workplaces after a process change or a change in the materials or equipment, being skillful in evidence-based decision-making as a means for response to the changing nature of the workplace hazards is important. Considering the characteristics and importance of occupational health services, and legal and juridical aspects of these services, observing the principles of professionalism is emphasized.

According to the outcomes-based framework of Can Meds [56], the matrix between tasks and expected roles of the providers of occupational health services was compiled. The results showed that in the axis of “exposure risk assessment”, performing tasks in the role of an evaluator has the highest importance for providers of occupational health services, in the axis of “controlling occupational hazards” consultant and evaluator roles were important, and in the axis of “fitness for work” and “health risk assessment” the people are prepared for playing the role of evaluator, screener and professional/expert. The educators are prepared to play the role of a therapist and professional/expert in the axis of “managing occupational diseases”, and the role of screener and professional/expert in “inter-organizational and inter-disciplinary relations and legal judgment” axis.

In this study, task- teaching–learning and evaluation methods matrix showed that various methods of teaching and evaluation are considered. In teaching methods, different interactive methods in small group, simulation and PBL settings based on inter-professional education was emphasized. The learners experience the exposure to problems similar to the real world and learn how to manage them. In addition, teaching in the real environment in inter-professional teams provides valuable opportunities for them to experience the situations which they will face in their future career. Evaluation methods of the learners’ competencies in performing their tasks, was designed in different levels of cognition, performance and attitude, using various tools.

The present study used the content validity methodology introduced by Lynn [27]. This method categorizes the framework of content validity into two stages including development and assessment. Recently, content validity methodology has gained special attention especially in developing instruments [24, 25, 57]. In this model, it is possible to compile content and structure and evaluate their validity. In this study, in the development stage, there was a possibility to extract the tasks from different sources such as literature review and expert opinion, which provided a complete content coverage of all tasks. In the next step, subtasks were determined for each task. In this step, the expert opinions in the panel helped us determine a set of essential subtasks by careful concentration on each task. General and specialized competencies were also paid attention to in this stage. In the next stage, tasks and subtasks were categorized according to Lynn's study [27]. This step helped structured categorization as well (convergence phase). Besides, the participants in the panel had the opportunity to re-evaluate the content coverage of the categories and be confident of the complete content coverage of the framework. In the second stage, the framework and tasks were assessed separately and validity indices were measured for each item and the whole framework.

Halek et al. similar to the present study used the content analysis method introduced by Lynn. They performed their study in three categories (evaluation, development, and judgment and quantification) and five stages. The stages included a literature review in two stages, an expert panel for their first evaluation of the instrument and a second and third evaluation in a workshop and the field [58]. They used only a literature review in the development phase and expert opinions were used in the second stage by assessing validity indices. They also used their instrument in the real environment to assess its applicability and practical use, but in the current study, expert opinion was used in the first stage as well to gain complete content coverage. Zamanzadeh et al. consistent with the present study, used content validity assessment in both stages of development and judgment, and the content validity indices were measured in the second stage [59]. In the current study, different sources (such as literature review and expert opinion) and methods (consensus-based methods such as the Delphi technique) were applied in the development stage. When there is a limitation of access to the participants, the Delphi method is suitable [60, 61], so in the current study, considering the participation of individuals from five different universities, the Delphi technique was used.

### Limitations

In this study, the tasks were compiled based on the different views of experts in one country. The limitation of number of participants in one environment is one of the limitations of the present study. Due to the differences in the domestic laws and guidelines in the occupational health field, this framework's generalizability to other environments is limited**.**

Advantages: To the best of our knowledge, this was the first study to develop an interprofessional task-based learning program and its related matrices in Iran which can be used for future studies in this field and also for curriculum development of the disciplines in which graduates are involved in providing occupational health services.

## Conclusion

The present study used a content validity study to develop a task framework and interprofessional task-based educational program by integrating interprofessional learning strategy and task-based learning in the occupational health field. Several tasks and subtasks categorized into seven main areas were extracted, developed, and evaluated. The extracted tasks were related to the future career of different learners in the occupational health team. The framework can facilitate the implementation of interprofessional education on different occasions such as in simulation and workplace-based learning situations as a road map.

## Data Availability

The datasets used and/or analyzed during the current study are available from the corresponding author upon reasonable request.
